# Role of the intercalated disc in cardiac propagation and arrhythmogenesis

**DOI:** 10.3389/fphys.2014.00404

**Published:** 2014-10-17

**Authors:** Andre G. Kleber, Jeffrey E. Saffitz

**Affiliations:** Department of Pathology, Beth Israel Medical Center, Harvard Medical SchoolBoston, MA, USA

**Keywords:** propagation velocity in heart, electrical-cell-to-cell coupling, intercalated disc, cardiac connexins, discontinuous propagation

## Abstract

This review article discusses mechanisms underlying impulse propagation in cardiac muscle with specific emphasis on the role of the cardiac cell-to-cell junction, called the “intercalated disc.”The first part of this review deals with the role of gap junction channels, formed by connexin proteins, as a determinant of impulse propagation. It is shown that, depending on the underlying structure of the cellular network, decreasing the conductance of gap junction channels (so-called “electrical uncoupling”) may either only slow, or additionally stabilize propagation and reverse unidirectional propagation block to bidirectional propagation. This is because the safety factor for propagation increases with decreasing intercellular electrical conductance. The role of heterogeneous connexin expression, which may be present in disease states, is also discussed. The hypothesis that so-called ephaptic impulse transmission plays a role in heart and can substitute for electrical coupling has been revived recently. Whereas ephaptic transmission can be demonstrated in theoretical simulations, direct experimental evidence has not yet been presented. The second part of this review deals with the interaction of three protein complexes at the intercalated disc: (1) desmosomal and adherens junction proteins, (2) ion channel proteins, and (3) gap junction channels consisting of connexins. Recent work has revealed multiple interactions between these three protein complexes which occur, at least in part, at the level of protein trafficking. Such interactions are likely to play an important role in the pathogenesis of arrhythmogenic cardiomyopathy, and may reveal new therapeutic concepts and targets.

## Introduction

The observation that the heart is composed of billions of individual cardiac myocytes but functions as a highly coordinated syncytial structure has fascinated scientists since the nineteenth century (Engelmann, 1877). In 1952, Weidmann used classical electrical cable analysis and showed that electrical current injected into a single cell in a Purkinje strand spread along a distance considerably larger than the length of the cell itself, thus suggesting the free movement of electrical charge from one cell to another (Weidmann, 1952). This finding, implicating low resistance pathways between cardiac myocytes, was later confirmed in ventricular muscle (Weidmann, 1966). Since this pioneering work, gap junction channels bridging the cytoplasm of neighboring cells, and forming pathways for cell-to-cell flow of electrical current and small molecules, have been characterized in detail at the genetic, cell biological, and biophysical levels of organization and function (Harris, 2001; Valiunas et al., 2005).

With respect to the physiological role of the cardiac cell-cell interface, the “intercalated disc,” the function and role of gap junctions, hosting channels composed of variety of connexins with regional specificity, has been the focus of many experimental and theoretical studies during the past decades (Kleber and Rudy, 2004). While the importance of gap junctions for cardiac function is not disputed, more recent work has shown that the intercalated disc also hosts a number of ion channels, including Na^+^ channels and K^+^ channels. Together with the proteins forming fascia adherens junctions and desmosomes (which interconnect actin filaments and intermediate filaments of adjacent cells), gap junction channels and ion channels form macromolecular protein complexes in the intercalated disc, each essential for cardiac function (Delmar and Liang, 2012; Delmar and Makita, 2012). Recent experimental studies suggest regulatory interactions between these functional complexes and their involvement in the pathogenesis of important cardiac diseases.

The goal of this review is to discuss (1) the role of gap junction channels and ion channels expressed in the intercalated disc in electrical propagation, and (2) interactions between these functional complexes and their role in disease.

## The cardiac connexins and electrical propagation

### The cardiac connexins Cx43, Cx40 and Cx45

In atrial and ventricular myocardium, gap junction channels are formed by three connexins, connexin43 (Cx43), connexin40 (Cx40) and connexin45 (Cx45) (Kanter et al., 1992; Davis et al., 1994). A fourth connexin has been described in the atrioventricular node in mice and humans (Cx30.2 and Cx31.9, respectively) (Bukauskas et al., 2006). Atrial myocardium gap junctions consist mainly of Cx43 and Cx40, ventricular myocardium of Cx43, and the specific ventricular conducting system of Cx40. Cx45 is present in the sinus and the atrio-ventricular nodes, and in small amounts in the atria and ventricles. Knowledge about the functional roles of Cx43, Cx40, and Cx45 in the cardiac intercalated disc has been derived mainly from experiments in heterologous cell systems and some questions remain unresolved. Common to all cardiac gap junction channels is the apposition and non-covalent binding of two juxtaposed connexons (each expressed in the intercalated disc by adjacent cells) to form a complete gap junction channel, and the composition of each connexon by six connexin molecules (Moreno, 2004; Cottrell and Burt, 2005). Pure homotypic and homomeric gap junction channels composed of Cx43 or Cx40 have relatively large electrical conductances (60–120 pS, and 175–210 pS, respectively) whereas Cx45 forms a small conductance channel of 30–40 pS (Moreno, 2004).

While it is often stated that the dominance of Cx43 in the ventricle and of Cx40 in the Purkinje system are responsible for the relatively high propagation velocities observed in these tissues, and that the presence of Cx45 contributes importantly to the low velocity in the atrio-ventricular node (for values see Kleber et al., 2011), the co-existence of Cx43 and Cx45 in the ventricles, and of Cx40, Cx43 and Cx45 in the atria, likely creates more complex biological interactions in which a connexin species present in relatively low amounts may have an important effect on conduction or modulate other aspects of cell-cell communication. For example, Cx45 has been shown to form heteromeric connexons with Cx43 (Moreno, 2004), and probably Cx40 (Desplantez et al., 2012) thereby reducing the unitary conductance of the channels. It has also been postulated that although Cx45 may not contribute much to intercellular electrical conductance by forming homomeric/homotypic channels, it could function as a modulator of gap junction size (Grikscheit et al., 2008).

In atrial myocardium Cx40 and Cx43 co-localize in the intercalated disc, but the role of mixed Cx43/Cx40 gap junction channels has not been clarified. In heterologous expression systems, it has been shown that Cx43 and Cx40 chemically aggregate, but the question of whether heteromeric connexons and heteromeric/heterotypic Cx43/Cx40 gap junction channels contribute significantly to intercellular current flow in heart remains disputed (Beyer et al., 2001; Cottrell and Burt, 2005). Another question of functional importance is whether expression and/or trafficking of Cx43 and Cx40 are coordinately regulated in atrial tissue. In human atrial tissue, for example, local expression levels of Cx43 and Cx40 affect local propagation velocity; high levels of Cx40 relative to Cx43 are inversely correlated with propagation velocity, whereas high levels of Cx43 relative to Cx40 correlate directly with local propagation velocity (Kanagaratnam et al., 2004). These results are in line with findings in cultured mouse atrial myocytes, in which genetic ablation of Cx40 is associated with *increased* propagation velocity and more Cx43 immmunosignal at the intercalated disc, whereas ablation of Cx43 leads to a decrease of propagation velocity and a concomitant reduction in Cx40 and Cx45 signal (Beauchamp et al., 2006; Desplantez et al., 2012). A further indication that atrial connexins are coordinately regulated comes from analysis of intercellular conductances in cells transfected with mutant Cx43 (Thibodeau et al., 2010). In this situation, co-expression of mutated Cx43 with wild type Cx43, or co-expression of mutated Cx43 with wild type Cx43 and wild type Cx40 produced a transdominant negative effect of the mutated Cx43 on intercellular conductance, indicating interactions between mutated Cx43 and wild type Cx43 and Cx40, possibly at the level of trafficking. Although basic mechanisms of connexin forward and retrograde trafficking have been defined recently (Smyth et al., 2010, 2012, 2014; Smyth and Shaw, 2012a,b), interactions between Cx43 and Cx40 and the resultant effects in cardiac disease remain largely unclarified.

### The effect on propagation of connexin compartmentation at the cell surface

Immediately after birth ventricular myocytes have a spindle-like shape and gap junction plaques are distributed at more or less regular intervals around the cell perimeter (Beauchamp et al., 2004). During development of the adult phenotype, gap junctions become concentrated at the cell border, and fewer junctions (~30%) are located in the lateral cell membrane (Hoyt et al., 1989). In an elegant theoretical study specifically addressing the effect of gap junction distribution on propagation (Spach et al., 2000), the authors compared *two real cell types*, small neonatal canine ventricular myocytes with neonatal gap junction pattern and large adult canine ventricular myocytes with typical adult end-to-end gap junction distribution pattern, to *two virtual cell types*, small neonatal myocytes with the adult gap junction distribution pattern and large adult myocytes with the neonatal pattern. This approach enabled the authors to separate the effects of cell size from the effects of gap junction distribution on propagation. The surprising result was that the pattern of gap junction distribution exerts only a minor influence on propagation, while cell size (as expected) has a strong influence on propagation velocity.

### The principle of electrical cell-to-cell coupling and cardiac propagation

**Figure 1:**
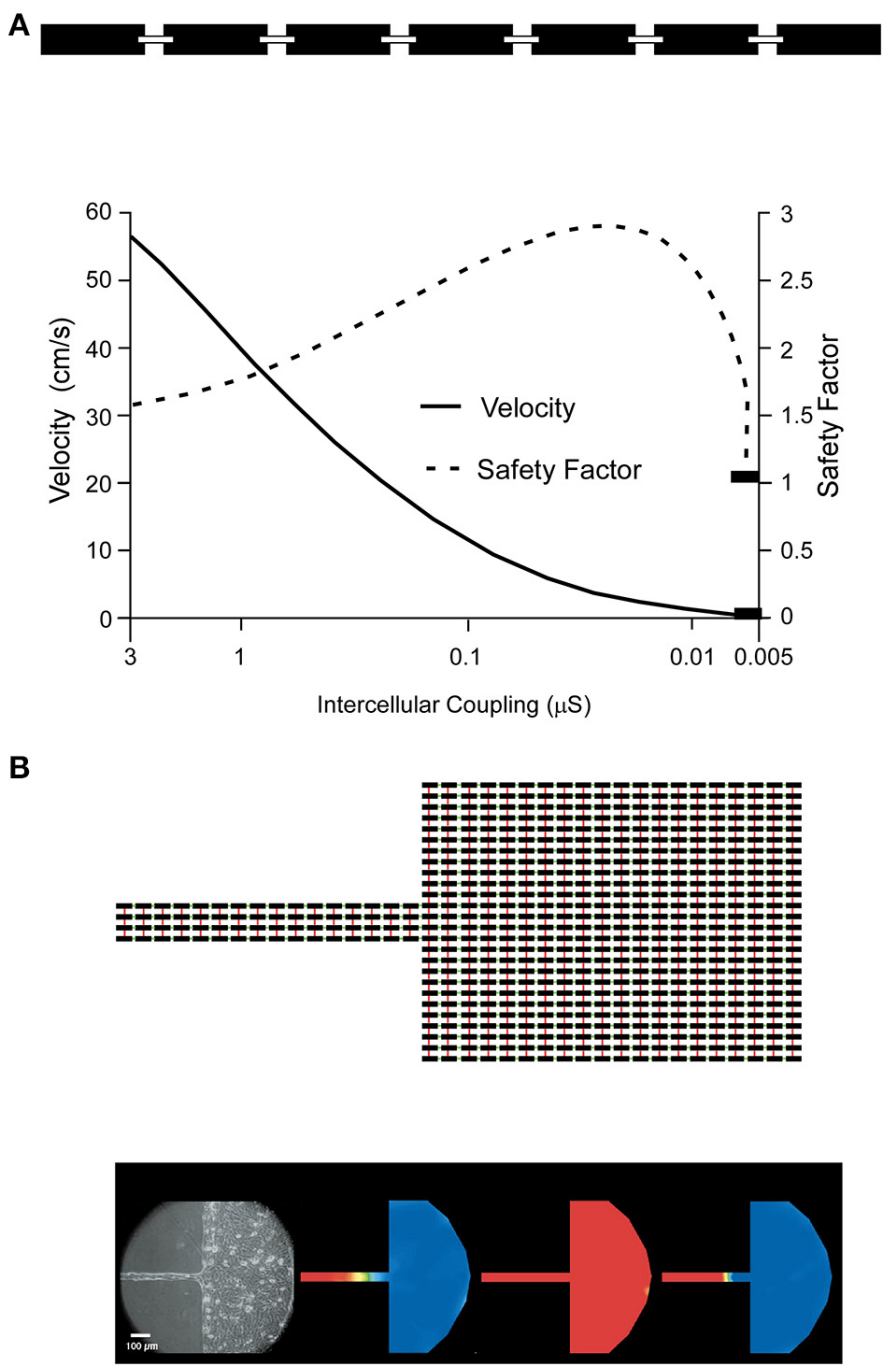
**Effect of cell-to-cell coupling on linear (A) vs. discontinuous (B) propagation: (A)** depicts schematically a linearly conducting chain of cells coupled by resistors representing the gap junctions (top). The lower part shows the dependence of propagation velocity and safety factor of propagation on cell-to-cell coupling, expressed as conductance in nS. For explanation see text. Reproduced from Shaw and Rudy (1997) with permission. **(B)** The equivalent electrical circuit of a discontinuous tissue structure is illustrated on top. Black rectangles correspond to cells linked by resistors in the x-axis direction (green) and y-axis direction (red). The bottom (left most part) shows experimentally determined propagation in a engineered cell culture of the shape depicted on top (neonatal rat ventricular myocytes). Right of the cell culture picture three propagation maps are coded in color: activation (presence of action potential) is coded in red, resting cells are coded in blue. Normal propagation (left color map) from the strand to the bulk of cells is blocked in the strand, because of source-sink mismatch. Partial uncoupling of the cells restores propagation across the strand and into the bulk (middle color map), whereas as full gap junction uncoupling of the bulk cells produces block at the expansion (right color map). From Rohr et al. (1997) with permission.

Insights into the roles of gap junctions and pathways of low electrical resistance between adjacent cells in propagation were derived originally from theories established in nerve (Hodgkin and Rushton, 1946). For propagation in a linear excitable structure with a homogeneous “intracellular electrical medium,” the square root of propagation velocity is proportional to the electrical resistance of the intracellular medium (so-called “square root relationship”) (Hodgkin, 1954; Tasaki and Hagiwara, 1957). In heart, the intracellular electrical medium is not homogeneous. Rather, the resistance of the cytoplasm is lower than the resistance of the gap junctions, and cytoplasmic propagation shows a finite conduction time (Fast and Kleber, 1993; Spach and Heidlage, 1995). Yet, for normal electrical coupling in heart and for small deviations thereof, the square root relationship is an approximate predictor of conduction behavior. At medium to high levels of electrical uncoupling, when the resistance of the intercellular barrier increases, the propagation behavior becomes discontinuous (Rudy and Quan, 1987). Discontinuous propagation in a linear strand of cells with uniform electrical uncoupling is resistant to propagation block, and a more than 100-fold increase in gap junction resistance may be needed for block to occur (Figure 1A). This behavior, demonstrated in theoretical studies, was confirmed in engineered strands of neonatal rat ventricular myocytes uncoupled by palmitoleic acid (Rohr et al., 1998), and in strands with complete (i.e., germ-line) genetic ablation of Cx43, in which only a low degree of residual electrical coupling is present due to Cx45 (Beauchamp et al., 2004).

For simple multicellular structures represented, for instance, by a linear strand of cardiac myocytes, this behavior can be explained by the effect of the coupling resistance on the so-called safety factor of propagation (Shaw and Rudy, 1997). The safety factor (SF) can be defined as the ratio of the electrical charge produced by an excited cell and the charge needed to depolarize the same cell. As long as the charge produced by excitation is greater than the charge needed for excitation (SF > 1), propagation is successful. The electrical charge produced by excitation (numerator in the SF formalism) must fulfill two functions which balance one another: it must change the charge at the membrane capacitance and thus produce the action potential, and it must drive the flow of electrical current across cell borders to excite cells downstream of the propagating wavefront. Importantly, increasing cell-to-cell electrical resistance homogeneously will slow propagation, but it will also enhance propagation safety (Shaw and Rudy, 1997) by limiting axial current being dissipated into the downstream cells. It will thus change the balance between the amount of charge flowing into the membrane capacitance and the amount of axial current flow, thereby promoting a higher and faster charge of the capacity and, accordingly, faster action potential upstroke (increase of *dV*/*dt*_max_ of the action potential). The fact that electrical uncoupling slows propagation (initially, as aforementioned with an increased propagation safety) is related to the decrease in axial current exciting the downstream cells. Initially this decrease causes slowing of the charge flowing into the membrane capacitance downstream and conduction slowing. Only when the degree of uncoupling becomes extreme does propagation safety decrease and block occurs (Shaw and Rudy, 1997).

While the safety factor principle, as defined by Shaw and Rudy, is very useful for understanding the general dependence of propagation on electrical coupling and the change in ion channel function (not discussed here) in linear structures, two further complexities warrant discussion: the discontinuous nature of cardiac architecture, and the effects of heterogeneity in expression of cardiac connexins with concomitant heterogeneity in electrical coupling.

### The interaction between the micro-structure of the myocardial cellular network and propagation

Normal cardiac myocardium shows a laminar arrangement of myocardial “sheet-like” structures wrapped around the left ventricle and bridged by small trabeculae at regular intervals (Legrice et al., 1995; Pope et al., 2008). These structural discontinuities of the myocardial network occur at a scale similar to the wavelength of electrical excitation (approximately 1 mm) and are predicted to affect the behavior of the electrical waves. Moreover, additional structural changes, caused for instance by fibrosis in pathological settings or with aging (Dolber and Spach, 1987), may enhance this interaction. In the context of this review, it is important to discuss the role of cell-to-cell coupling as modulator of propagation at structural discontinuities, as shown by several theoretical and experimental studies. The ability to pattern 2-dimensional multicellular cardiac structures in culture to produce distinct and reproducible geometrical shapes offers the opportunity to study the effect of cellular network structure on propagation, and to unveil the biophysical behavior of such structures in theoretical studies using equivalent simulated tissue shapes (Rohr et al., 1991).

A typical engineered structure is represented by a small fiber emerging from, or leading into, a large bulk of tissue (so-called “geometrical expansion”). The structural mismatch between the small fiber and the large bulk leads to a corresponding asymmetrical mismatch in electrical propagation. When the small fiber leads into a large bulk, axial current produced by the small amount of cells in the strand has to excite the large bulk. The expansion between the strand and the large mass causes axial electrical current to disperse (current sink), thus, causing current density to decrease. This dispersion causes a local delay in propagation and, in the extreme case, propagation block (Fast and Kleber, 1995a,b; Rohr et al., 1997). In the opposite direction, propagation remains safe, because the large bulk of tissue has only to excite the small mass of cells in the narrow fiber. Thus, the geometrical expansion causes propagation block to be unidirectional. Unidirectional block has been recognized as a prerequisite for initiation of circus movement and re-entry since the beginning of the last century (Mines, 1913). Similar phenomena occur at other types of structural discontinuities such as pivot points and isthmuses, and obey the same biophysical principles (Cabo et al., 1996, 1998; Fast et al., 1996; Kleber and Rudy, 2004).

Importantly, discontinuities in propagation, as described above, are very sensitive to the function of ion currents responsible for excitation and to the state of underlying electrical cell-to-cell coupling. Partial uncoupling of cells at a geometrical expansion produces resistance to dissipation of local current at this site and to a decrease in the current sink (Figure 1B). Reduced dispersion of axial current protects the impulse from being blocked at the expansion, a phenomenon that has been demonstrated in experimental and theoretical studies (Fast and Kleber, 1995a,b; Rohr et al., 1997). As a consequence, application of agents causing partial uncoupling of cells can reverse the unidirectional block and allow bidirectional propagation. In whole tissue this is predicted to decrease the probability of initiation of re-entry. In addition to the state of electrical cell-to-cell coupling, the state of the ion channels is an additional player affecting propagation at geometrical expansions, pivot points, and isthmuses. For example, the fact that impulses are delayed locally changes the contributions of the two charge carriers in excitation, the Na^+^ inward current and the L-type Ca^2+^ inward current, to successful propagation. At a geometrical expansion the downstream cells are excited with a delay relative to the upstream cells. Accordingly the upstroke of the downstream action potential may occur at a time when the upstroke of the upstream action potential is nearly or fully complete. As a consequence the inward Na^+^ current of the upstream cells is “too fast” to excite the downstream cells, and the excitatory charge is delivered instead by the slower L-type Ca^2+^ current. In other words, with increasing discontinuity the flow of L-type Ca^2+^ current becomes increasingly more important as the charge carrier for excitation (Shaw and Rudy, 1997). Proof of this behavior, which has been shown in several theoretical studies (Kleber and Rudy, 2004), is given by the observation, shown in Figure 1, that anterograde propagation across a geometrical expansion can be blocked by a Ca^2+^ channel blocker, depending on whether the source-to-sink mismatch is large enough and leads to a local delay >2 ms (Rohr and Kucera, 1997). This effect depends on the underlying state of cell-to-cell coupling, because partial uncoupling will decrease the degree of source-to-sink mismatch for a given structural discontinuity.

Taken together, these observations demonstrate that (1) microscopic tissue structure, (2) expression of ion channels and associated flow of depolarizing currents (Na^+^ current and L-type Ca^2+^ current), and (3) cell-to-cell electrical coupling interact to affect local propagation and define local propagation and risk of unidirectional propagation block in tissues with a discontinuous structure such as occurs in the heart. Since microscopic cardiac structure may vary between individuals and may be affected by fibrosis associated with disease and age, it becomes difficult to accurately predict arrhythmogenesis and its prevention by drugs in any individual situation. Experimental and theoretical studies offer more of a general understanding, and the potential arrhythmogenic effect of changes in cell-to-cell coupling cannot be considered independently of changes in the other variables in this highly interactive system.

### Electrical impulse propagation in presence of very low and heterogeneous connexin immunosignal: does ephaptic impulse transmission play a role?

Experiments involving conditional cardiac myocyte-specific knock-out of Cx43 in mice have shown that a marked decrease in Cx43 expression is necessary to produce ventricular arrhythmias. In the first such study (Gutstein et al., 2001a), in which genetic ablation of Cx43 by a Cre/loxP system decreased total myocardial Cx43 protein by 86–95% associated with a marked reduction in Cx43 immunosignal, electrical propagation velocity was reduced by only ~50% and, in contrast to the situation with germ-line knock-out of Cx43, mortality was delayed by 15–30 days. In two subsequent studies, cardiac myocyte-specific genetic approaches were used to produce a progressive decrease in Cx43 expression with age (Danik et al., 2004) or to create a mosaic of ventricular tissues showing either normal or absent Cx43 expression (Gutstein et al., 2001b). In the first model, significant propagation slowing occurred only when the amount of Cx43 immunosignal decreased by ≥50%, and malignant ventricular arrhythmias were initiated if Cx43 immunosignal was ≤18%. In the second model, mosaic expression caused much more irregular electrical excitation patterns and marked arrhythmogenesis, suggesting that not only the average degree of ventricular Cx43 expression but also the distribution patterns and scale of heterogeneity might be important. In a subsequent study, intercellular conductance measured by dual whole cell voltage clamp in pairs of adult rat ventricular myocytes with genetic Cx43 ablation was dramatically reduced from 588 to 10 nS (Yao et al., 2003). Since impulse propagation was reduced by only 50% in tissue composed of the same cells (with conditional cardiac-specific Cx43 knockout associated with a marked decrease of Cx43 immunosignal), these observations raised the question of whether propagation of the cardiac impulse could occur in the absence of electrical cell-to-cell coupling. In fact, this phenomenon, so-called ephaptic impulse transmission, due to local circuit current at the intercalated disc, had been postulated in the 1960s (Sperelakis et al., 1960) and proposed as an alternative to the role of gap junction channels (Weidmann, 1993).

Theoretical studies have investigated the possible role of ephaptic impulse transmission (also termed “field effect transmission”) in myocardium using computer models that take into account the presence of Na^+^ channels at the intercalated disc (Kucera et al., 2002; Mori et al., 2008; Veeraraghavan et al., 2014). The key difference in modeling an electrical equivalent circuit for ephaptic transmission relative to earlier models is the focus on the pool of Na^+^ channels located within the intercalated disc, which in biophysical terms, represents a narrow cleft with an electrical resistance higher than the normal extracellular resistance. As a consequence, excitation of the upstream cell by inward flow of Na^+^ current establishes a voltage gradient between the cleft space and the normal extracellular space, which acts to depolarize the cleft space and the Na^+^ channels in the downstream cell facing the cleft. The excitatory effect on the downstream cell, which theoretically may occur in the absence of resistive coupling by gap junctions, depends critically on the cleft width (determining cleft resistance) and the extent to which Na^+^ channels are clustered in the intercalated disc. These models also suggest that Na^+^ ions accumulate with narrowing cleft width, a phenomenon that decreases the electrochemical gradient for Na^+^ and is, therefore, expected to decrease Na^+^ current (Kucera et al., 2002; Mori et al., 2008).

The hypothesis that ephaptic transmission occurs in the heart under conditions of marked cell-to-cell uncoupling was put forward as an alternative idea to explain why propagation velocity is only moderately reduced when Cx43 levels are apparently very low, such as occurs in mouse hearts following conditional cardiac myocyte-specific knockout of Cx43. However, its existence has never been proven experimentally. In pairs of cultured neonatal myocytes the existence of ephaptic transmission has been ruled out (Beauchamp et al., 2004). In pairs of ventricular myocytes with germline genetic ablation of Cx43 (i.e., not dependent on the action of Cre recombinase) there is residual electrical coupling (about 5% of normal) due to the presence of gap junction channels formed by Cx45. If these cells are chemically uncoupled, electrical impulse transfer is immediately and reversibly interrupted, while the cells remain excitable by field stimulation, demonstrating that in this setting ephaptic transmission cannot substitute for resistive coupling by gap junctions (Beauchamp et al., 2004).

**Figure 2:**
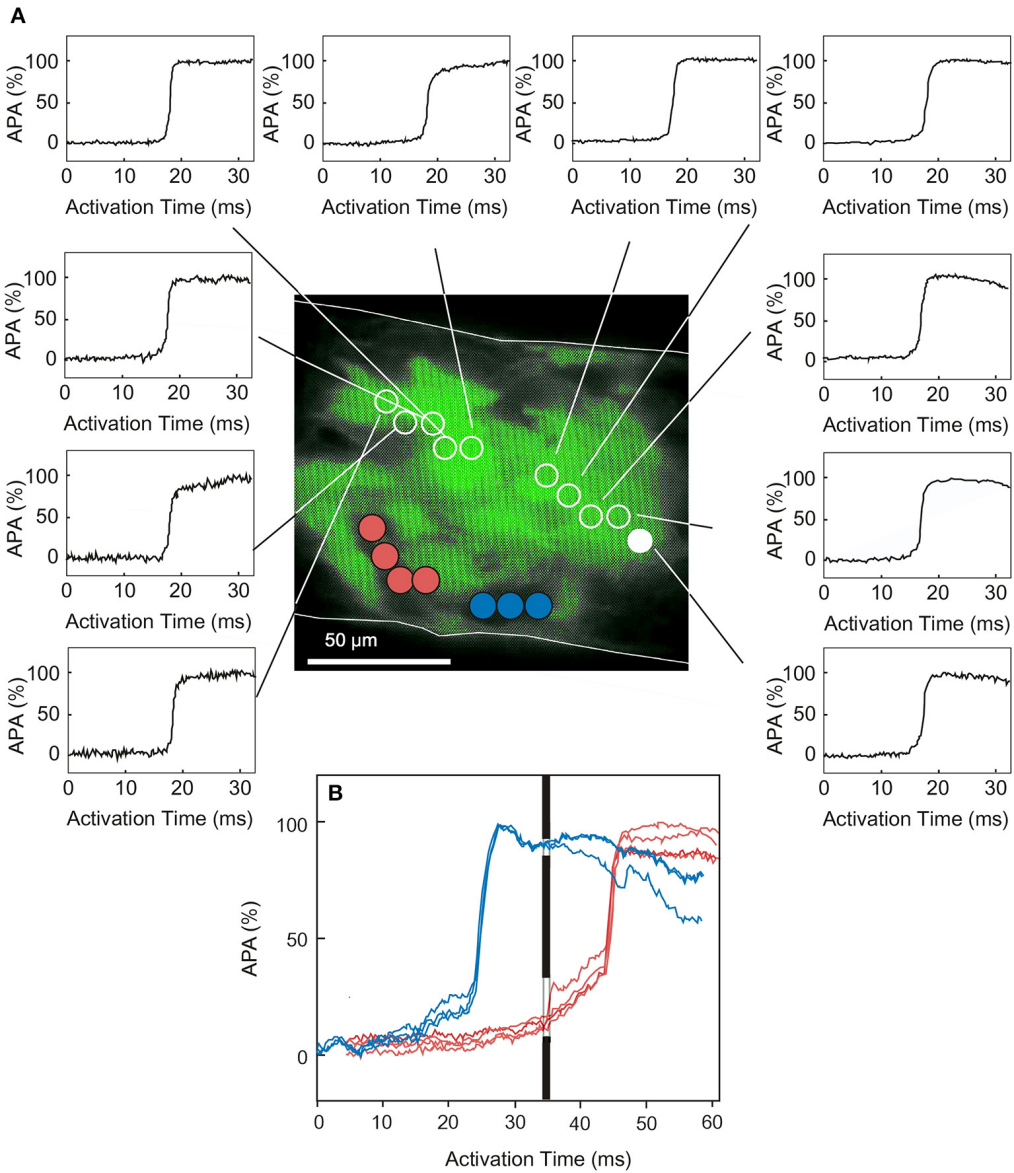
**Propagation in engineered cardiac tissue with heterogeneous expression of Cx43. (A)** An engineered cardiac strand is produced by 50–50 mixtures of wild type cells (GFP-labeled) and cells with genetic ablation of Cx43 (no GFP-labeling). Electrical stimulation of this strand produces rapid meandering propagation across the Cx43 expressing cell cluster. The action potentials in **(B)** compare excitation of the area with Cx43 deletion (colored action potential) with the first and last action potentials of the Cx43 expressing cluster (which, at this time scale, appear to be nearly superimposed). This comparison illustrates a marked pre-delay and delay in excitation of the areas not expressing Cx43 with respect to the wild type cluster. However, all cells of the strands are eventually excited. From Beauchamp et al. (2012) with permission.

An attempt to experimentally elucidate the mechanism of impulse propagation in ventricular tissue with inhomogeneous reduction in connexin distribution has been made by (1) quantifying the relationship between the amount of Cx43 at the intercalated disc (as measured by immunohistochemistry) and intercellular electrical conductance across the intercalated disc (McCain et al., 2012), and (2) engineering strands of neonatal mouse ventricular myocytes composed of fixed mixtures of wild type myocytes and cells with genetic Cx43 ablation (Beauchamp et al., 2012). Using this approach, it was first shown that the amount of Cx43 quantified immunohistochemically correlates linearly with electrical conductance, but low levels of conductance can be measured under conditions in which Cx43 signal is below the level of detection using conventional indirect immunofluorescence methods (McCain et al., 2012). In other words, the absence of detectable Cx43 immunosignal does not exclude residual electrical coupling and impulse transmission. In another study (Beauchamp et al., 2012), recently supported by theoretical work (Prudat and Kucera, 2014), it was shown that in tissue with heterogeneously distributed Cx43 expression, fast propagation can be maintained by meandering propagation involving cells with higher Cx43 levels (Figure 2). Marked propagation slowing is observed only when the proportion of Cx43-null cells falls below 50%. Moreover, the overall amount of apparent Cx43 immunosignal in such tissues markedly underestimates the percentage of cells expressing normal levels of Cx43 because no Cx43 immunofluorescence is detectable at boundaries between Cx43 expressing myocytes and cells with genetically ablated Cx43 expression (Beauchamp et al., 2012). In conclusion, the observation of relatively preserved propagation at low and inhomogeneous levels of Cx43 expression can be accounted for by cell-to-cell impulse transmission solely involving gap junction channels. The existence of ephaptic transmission as an additional modulator of cardiac impulse propagation has yet to be clearly demonstrated experimentally, although it can be simulated theoretically within narrow limits of specific variables. More sophisticated experimental studies will be required to clarify this point.

## Interactions between desmosomal proteins, ion channels and connexins at the intercalated disc

In the previous paragraphs, this review has focused on the role of gap junctions in impulse transmission and propagation. Ion channels were invoked in the discussion insofar as they might theoretically play a role in cell-to-cell transmission of the electrical impulse. Now, we will discuss mutual interactions between (i) desmosomal and fascia adherens proteins, (ii) connexins and (ii) ion channels expressed in the intercalated disc. New insights into such interactions have emerged recently, stimulated in large part by work to understand mechanisms in arrhythmogenic cardiomyopathy. It thus forms an interesting link to translational medicine and is an example how findings in translational science can feed back to the understanding of basic mechanisms of cardiac function. It mostly involves defining the effect of genetic ablation of components of one of these protein complexes or the use of cells transfected with mutant proteins implicated in human disease, and the subsequent association of the findings in animal models with the human phenotype.

An important principle underlying this work is that major classes of ion channels responsible for generating the cardiac action potential are partitioned in different membrane compartments such as the T-tubular invaginations, the (lateral) surface membrane and the intercalated discs (Cohen, 1996; Kucera et al., 2002; Maier et al., 2004; Petitprez et al., 2011; Milstein et al., 2012). The specificity of Na^+^ channel partitioning has been further explained by observations that channels in specific locations are associated with specific regulatory proteins. This includes dystrophin in the case of Nav1.5 channels at the lateral membrane, and the MAGUK protein SAP97 for Nav1.5 at the intercalated disc (Petitprez et al., 2011). It has also been shown that SAP97, which has PDZ binding motifs for several ion channels, is an important regulator of the intercalated disc fractions of Nav1.5 and Kir2.1 (Milstein et al., 2012). At the same time, recent studies involving knock-in of Nav1.5 channels lacking the PDZ binding motifs have implicated a more complex role for SAP97 involving localization of ion channels at the lateral membrane (Shy et al., 2014). Within the intercalated disc Nav1.5 channels have been shown to closely localize with Cx43 in a region around the gap junction plaque termed the perinexus (Rhett et al., 2012).

Recent studies have identified mechanistic links between proteins of the desmosome, Nav1.5 and Cx43. For example, silencing of plakophilin-2, an armadillo repeat protein contributing to the protein anchor of desmosomal cadherins, leads to a marked decrease in Na^+^ inward current via Nav1.5 at the intercalated disc (Sato et al., 2009). Interactions between Cx43 and Nav1.5 expression at the intercalated disc are also suggested by observations that germline ablation of Cx43 in cultured neonatal rat atrial myoctes leads to a nearly 50% decrease in whole cell Na^+^ current (Desplantez et al., 2012). Ankyrin-G, a polypeptide involved in surface expression and targeting of membrane channels, has been shown to affect plakophillin-2 and Nav1.5 expression at the intercalated disc (Sato et al., 2011; Makara et al., 2014). The role of ankyrin-G in the regulation of Cx43 is less clear. While silencing of ankyrin-G decreased Cx43 and intercellular electrical conductance (Sato et al., 2011), no effect of cardiospecific knock-out of ankyrin-G on Cx43 was observed (Makara et al., 2014). Taken together, these observations highlight important, although as yet incompletely understood, interactions among regulatory proteins at the intercalated disc, desmosomal proteins, connexins and ion channels (Nav1.5 and probably others) at the intercalated disc which likely play a significant role in human heart disease.

Additional knowledge about these interactions has come from work defining the molecular pathology of arrhythmogenic cardiomyopathy, a disease in which approximately 60% of cases have (mainly dominant) mutations in desmosomal proteins (plakoglobin, desmoplakin, plakophilin-2, desmocollin-2 and desmoglein-2) (Saffitz et al., 2009; Sen-Chowdhry et al., 2010). For reasons not well understood, the majority of these cases are associated with a decrease in the amount of plakoglobin (γ-catenin) in the intercalated disc and a concomitant decrease in Cx43, raising the possibility that plakoglobin might play a central role in disease pathogenesis (Asimaki et al., 2009). Reductions in the levels of desmosomal plakoglobin and Cx43 at the intercalated disc apparently occur in the presence of normal cellular levels of these proteins, thus implicating a protein trafficking defect as an underlying mechanism. Arrhythmogenic cardiomyopathy is also associated with a marked decrease in Nav1.5 expression, as inferred from experiments involving cultures of neonatal rat ventricular myocytes transfected to express mutant forms of plakoglobin or plakophilin-2 known to cause the human disease. In both situations, a ≥50% decrease in Na^+^ inward current has been observed (Asimaki et al., 2014).

A potentially important insight into the underlying molecular interactions came from discovery of a drug in a high throughput chemical screen in zebrafish. This small molecule, SB216763, annotated as an inhibitor of glycogen synthase kinase-3β (GSK-3β), prevents the development of cardiac failure in a fish model of arrhythmogenic cardiomyopathy and also prevents changes in the cardiac action potential and decreased currents flowing through Nav1.5 and Kir2.1 in isolated zebrafish ventricular myocytes. SB216763 also blocked expression of disease-related changes in cultured neonatal rat myocytes transfected to express known disease alleles (Asimaki et al., 2014). Furthermore, abnormal subcellular distribution of SAP97 is reversed by SB216763 in cardiac myocytes derived from pluripotent stem cells of 2 human probands carrying plakophilin-2 mutations. The exact role of GSK-3β and its inhibition in the disease phenotype is currently under investigation. Nevertheless, our current knowledge of disease mechanisms in arrhythmogenic cardiomyopathy suggests that expression of desmosomal proteins, connexins and at least two important ion channels (Nav1.5 and Kir2.1) are all regulated interactively at the level of trafficking.

## Summary: cell-to-cell coupling, arrhythmogenesis and propagation

In the classical literature, the effects of changes in cell-to-cell coupling are mostly discussed using simple models of linear propagation. In such models, a decrease in cell-to-cell coupling will reduce propagation velocity and, accordingly, decrease the wavelength of excitation. As a consequence an increase in the likelihood of re-entry of circulating excitation is to be expected. This review attempts to summarize and discuss both the classical and contemporary literature which, taken together, reveals a much more complex role for cell-to-cell coupling in normal physiology and arrhythmogenesis. These complexities involve heterogeneous expression of connexins, the structure of the cellular network and expression of ion channels in the intercalated disc all being interactively affected by common regulators of expression. Moreover, disease phenotypes are likely to involve changes in multiple proteins affecting electrical propagation. This complexity of the arrhythmogenic substrate makes it difficult to exactly predict the exact impact of changes in cell-to-cell coupling as an event isolated from all other factors. At the extreme—as discussed in this review—electrical uncoupling may weaken the effect of a discontinuous structure and actually stabilize propagation. Theoretical simulation of these complex interactions including the multitude of variables is certainly a means to improve our understanding of the role in arrhythmogenesis.

The fact that a single disease may produce hundreds of changes at the level of functional proteins, which may be arrhythmogenic in concert, also casts doubt on the concept that targeting a single peripheral protein, as only one of the multitude of variables, will effectively reduce arrhythmogenesis. Instead so-called “upstream drugs” targeting an important common pathway of dysregulation and reversing a pathological phenotype are likely to be more effective. The discovery of an apparent GSK-3β inhibitor as a substance globally rescuing the arrhythmogenic cardiomyopathy phenotype supports this concept.

### Conflict of interest statement

Kleber: Consultant, Schiller Inc., Baar, Switzerland. The authors declare that the research was conducted in the absence of any commercial or financial relationships that could be construed as a potential conflict of interest.
